# Synergistic strategies of *Salinivibrio kushneri* immobilization on biochar alginate microspheres for the removal of phenol from aqueous solution modeling approaches

**DOI:** 10.1038/s41598-025-20677-4

**Published:** 2025-10-01

**Authors:** Mohammed T. M. H. Hamad, Walaa S. Mohamed

**Affiliations:** 1 Microbiology Department, Central Laboratory for Environmental Quality Monitoring (CLEQM), National Water Research Center (NWRC), Cairo, Egypt; 2 Biology and Environmental Indicators Department, Central Laboratory for Environmental Quality Monitoring (CLEQM), National Water Research Center (NWRC), Cairo, Egypt

**Keywords:** Phenolic pollution, Immobilized bacteria, Biochar, Response surface methodology, Kinetic studies, Isotherm studies, Microbiology, Environmental sciences

## Abstract

Phenolic pollution poses a significant environmental issue due to its toxicity and persistence in aquatic ecosystems. This study investigates an innovative approach utilizing immobilized Salinivibrio kushneri within alginate and water hyacinth-derived biochar microspheres for the removal of phenol from aqueous solutions. Bacterial strains with high phenol-degrading capacity were isolated, and the best-performing strain was identified by 16 S rDNA sequencing (GenBank accession no. PQ836117). Two biosorbents were evaluated: Bacteria-Alginate Microspheres (BAMs) and Bacteria-Loaded Biochar-Alginate Microspheres (BLBAMs). The BLBAMs outperformed BAMs, achieving phenol removal rates over 87%. This maximum efficiency was achieved under optimal conditions of pH 7, 30 h contact time, and a phenol concentration of 51.76 mg/L. Kinetic and isotherm modeling indicated that the adsorption process was driven by chemisorption and followed the Langmuir model, with a monolayer adsorption capacity of 53.19 mg/g. The pseudo-second-order model (*R*^2^ = 0.999) provided the best fit to the data. The study demonstrates that integrating microbial activity with water hyacinth-derived biochar provides a sustainable and cost-effective method for phenol removal, supporting scalable bioremediation strategies for wastewater treatment.

**Supplementary Information:**

The online version contains supplementary material available at 10.1038/s41598-025-20677-4.

## Introduction

 Water resources are increasingly under pressure from intensive urbanization, rapid industrial growth, and population expansion worldwide. Among organic contaminants, phenolic compounds are of particular concern due to their widespread industrial applications, including oil refining, pharmaceuticals, agrochemicals, and paint production, and their frequent release into municipal and industrial effluents. Such discharges cause severe pollution of aquatic ecosystems and drinking water supplies^1,2^. Furthermore, during conventional disinfection processes, chlorination of phenol-contaminated water can generate chlorophenols, which are more toxic, recalcitrant, and environmentally persistent^3^. Phenol is a hazardous pollutant with high solubility and low biodegradability, making it difficult to eliminate from aquatic systems. Even at concentrations as low as 1 mg/L, phenol is toxic to aquatic organisms, and levels exceeding 10 mg/L are frequently lethal. This acute toxicity, combined with the formation of even more persistent chlorophenols during chlorination, underscores the urgency of developing efficient and sustainable removal strategies^1^. A wide range of physicochemical treatment methods, including solvent extraction, adsorption on activated carbon, advanced oxidation, and ion exchange, have been extensively studied for phenol removal. While effective under controlled conditions, these approaches face critical drawbacks such as high operational costs, secondary pollutant generation, limited reusability of adsorbents, and poor adaptability to complex wastewater matrices^4–6^. For instance, activated carbon, though widely used, exhibits declining efficiency after multiple cycles of use, while chemical oxidation can generate harmful by-products. These challenges highlight the urgent need for cost-effective, sustainable, and environmentally friendly alternatives. In recent years, biological methods notably microbial degradation and biosorption have gained momentum as promising alternatives. Immobilized microbial cells are widely recognized for maintaining high cell densities, enhancing degradation efficiency, and reducing cell washout in continuous bioreactors. However, numerous studies have documented persistent challenges with immobilization, such as poor mechanical strength of carriers, insufficient microbial adhesion, and instability under wastewater conditions^2,7,8^. While alginate remains a widely used natural polymer due to its biocompatibility and ease of gelation, its low durability and limited porosity hinder long-term performance. To overcome these shortcomings, recent studies have increasingly investigated biochar-based carriers, which provide large surface areas, abundant functional groups, and protective niches that enhance both adsorption and microbial colonization^6,9,10^. In particular, biochar derived from water hyacinth (*Eichhornia crassipes*) combines high porosity and surface activity with environmental sustainability by converting an invasive aquatic weed into a functional biosorbent, aligning pollutant remediation with circular economy principles^11^. To overcome these material limitations, the present study introduces bacteria-loaded biochar–alginate microspheres (BLBAMs) as a novel, efficient, and sustainable strategy for phenol removal. Biochar has been widely studied as an adsorbent and microbial support material, with diverse feedstocks including sugarcane bagasse^12^, coconut shells^13^, palm shells^14^, and modified *Hevea brasiliensis* seed shells^15^. Leaf-derived biochars, such as *Phoenix dactylifera* leaves, have also demonstrated promising adsorption capacities^16^, while advanced composites like graphene oxide PNIPAM^17^ and biochar–geopolymer hybrids^9^ illustrate the versatility of biochar-based systems. Against this background, water hyacinth (*Eichhornia crassipes*) offers distinctive advantages: high carbon content, low ash yield, and highly porous structures upon pyrolysis, making it particularly effective for microbial immobilization and phenol adsorption. Moreover, utilizing water hyacinth provides an ecological benefit by managing an invasive aquatic weed and promoting waste-to-resource circularity^11^. Despite these advances, the integration of biochar and alginate for microbial immobilization in phenol removal has been rarely explored, and the use of invasive aquatic weeds such as water hyacinth as sustainable biochar precursors remains largely underutilized. Furthermore, no previous studies have reported the incorporation of *Salinivibrio kushneri*, a phenol-degrading bacterium, within a biochar–alginate matrix. To address these gaps, the present study develops and evaluates bacteria-loaded biochar–alginate microspheres (BLBAMs) derived from water hyacinth, which uniquely combine alginate’s biocompatibility, biochar’s high porosity and stability, and *Salinivibrio*’s biodegradation capacity. This synergistic design offers a novel, durable, and environmentally sustainable strategy for efficient phenol removal while simultaneously valorizing an invasive aquatic resource within a circular economy framework.

## Materials and methods

### Chemicals

All chemicals used in this study were of analytical grade and obtained from Merck (Egypt), with the following purities: phenol (≥ 99%), sodium alginate (≥ 98%), calcium chloride (CaCl₂) (≥ 95%), magnesium sulfate heptahydrate (MgSO₄·7 H₂O) (≥ 98%), ammonium sulfate ((NH₄)₂SO₄) (≥ 99%), potassium dihydrogen phosphate (KH₂PO₄) (≥ 99%), disodium hydrogen phosphate dodecahydrate (Na₂HPO₄·12 H₂O) (≥ 98%), and acetonitrile (HPLC grade, ≥ 99.9%) for phenol quantification. Sodium alginate was chosen due to its biocompatibility, non-toxicity, and ability to form hydrogels, making it suitable for microbial immobilization. Calcium chloride served as the crosslinking agent to induce ionic gelation of alginate, thereby producing stable microspheres. Magnesium sulfate, ammonium sulfate, and phosphate salts were included as essential nutrients and buffering agents to support bacterial growth and maintain a stable pH during experiments. Acetonitrile was used as the mobile phase solvent in HPLC analysis of phenol. Phenol stock solutions were prepared by dissolving the required amounts of phenol in 1 L of distilled water and subsequently diluting to obtain different concentrations.

### Isolation and screening of bacterial strains for phenol biosorption

Bacterial strains were isolated from wastewater samples obtained from a treatment facility in Cairo, Egypt. These isolates were subsequently cultured on nutrient agar plates. The isolated strains were then sub-cultured in a nutrient broth (NB) medium at 37 °C with shaking at 160 rpm for 24 h to promote bacterial growth. After incubation, the biomass was harvested by centrifugation at 4000 rpm for 5 min. The resulting cell pellet was suspended in a fresh NB medium to form a concentrated cell suspension and incubated for 72 h^18^. For enrichment, the bacterial suspensions were transferred to a mineral salt medium (MSM) with the following composition per liter: 0.9 g of KH_2_PO_4_, 6.5 g of Na_2_HPO_4_·12H_2_O, 0.2 g of MgSO_4_·7H_2_O, and 0.4 g of (NH_4_)_2_SO_4_ supplemented with phenol at increasing concentrations (25–150 mg/L) to selectively enrich phenol-degrading strains. Only strains that survived and grew at higher phenol concentrations were retained for further biosorption evaluation.

### Molecular identification

Isolates showing more than 80% phenol removal at an initial concentration of 100 mg/L were selected for further study. In addition, screening criteria included rapid growth kinetics in phenol-containing media, tolerance to elevated phenol concentrations, stability of degradation efficiency over successive subcultures, and robust morphological characteristics. Based on these combined criteria, the isolate identified as *Salinivibrio kushneri* was selected for incorporation into the BLBAM system.

The selected bacterial isolate was identified using 16S rDNA sequencing. The genomic DNA of the isolated strains was extracted using a specialized kit (B518263-0050, 50 times/box, Shanghai Biotech, China) according to the manufacturer’s guidelines. After purification, agarose gel electrophoresis was performed with a 0.8% agarose gel in 100 mL of 1× electrophoresis buffer (prepared from 10× TBE: Tris-Borate EDTA, containing 108 g/L Tris-base, 55 g/L boric acid, and 40 mL of 0.5 M EDTA at pH 8). The purified genomic DNA was loaded with a stop-loading solution along with an appropriate DNA marker ladder. PCR was conducted using a gradient Robocycler 96 (Stratagene, USA) with a reaction mixture that included Master Mix (containing 2.5 U Taq DNA polymerase, 200 µM of each dNTP, and 1× Qiagen PCR buffer), 100 pmol of each primer (27F: 5’-AGAGTTTGATCCTGGCTCAG-3’ and 1492R: 5’-GGCTACCTTGTTACGACTT-3’), and 200 ng of DNA. The PCR cycling conditions were denaturation at 94 °C for 30 s, annealing at 55–60 °C for 25–30 s, and extension at 72 °C for 30–50 s. DNA sequencing was performed using the Cy5/Cy5.5 Dye Primer Sequencing Kit^19^. The sequencing results were analyzed by comparing them with entries in the gene bank. (https://www.ncbi.nlm.nih.gov/genbank/ ).

### Preparation of bacteria-alginate microspheres (BAMs)

After enrichment of the best absorbent bacterial isolate in broth medium, bacterial cells were harvested through centrifugation at 4000 rpm for 5 min and washed twice with potassium phosphate buffer (KH₂PO₄). Immobilization was achieved using a 2% (w/v) sodium alginate solution, as described by ^7^. A 2% (v/v) inoculum of bacterial cells was mixed into the sodium alginate solution. This mixture was extruded and gradually added dropwise using a 5 mL syringe from a height of 50 cm into a 2% (w/v) supersaturated calcium chloride (CaCl₂) solution, with intervals of 2–3 s between drops. The resulting beads were soft and elastic. The mechanical integrity of the prepared BAMs was assessed using a texture analyzer (TA.XT Plus, Stable Micro Systems, UK) equipped with a 5 mm cylindrical probe. Each microsphere was compressed at a constant speed of 1 mm/s, and the distance at which visible structural deformation occurred was recorded^20^. On average, the BAMs exhibited deformation at 0.84 ± 0.06 mm, based on triplicate measurements. This deformation threshold indicates that the microspheres possess adequate elasticity and structural stability to withstand physical stress during handling and application in aqueous phenol biosorption experiments.

### Preparation of water hyacinth-derived Biochar

Biochar was prepared from water hyacinth (*Eichhornia crassipes*), a fast-growing aquatic plant known for its high carbon content and strong adsorption capabilities. Water hyacinths were collected from the delta region of Egypt. The plant was then thoroughly rinsed with running water to remove dirt and impurities and left to dry in the sun for three days in an open location to reduce moisture content. Following this, the stems and leaves were separated and dried in an oven at 50 °C for 24 h ^21^. After drying, the stems and leaves were crushed and sieved using a 425 μm granulometric sieve. Subsequently, the dried water hyacinth was pyrolyzed at 600 °C for 1 h in a muffle furnace. This step was essential for optimizing carbonization, enhancing porosity, and improving the biochar’s ability to adsorb contaminants. After pyrolysis, the biochar was allowed to cool naturally inside the furnace to prevent oxidation and then stored in an airtight container to preserve its structure and properties^22^.

### Preparation of bacteria-loaded biochar-alginate microspheres (BLBAMs)

A 2% (v/v) bacterial suspension (OD₆₀₀ ≈ 1.0, corresponding to approximately 1 × 10⁸ CFU/mL) was prepared in sterile nutrient broth and added to 5 g of sterilized biochar in a 100 mL total volume. The mixture was incubated at 160 rpm and 37 °C for 24 h to facilitate bacterial attachment to the biochar surface. The mixture was incubated at 160 rpm for 24 h to allow the bacterial cells to adhere to the biochar’s surface and penetrate its pores. Following incubation, the bacteria-led biochar was added to a 2% (w/v) sodium alginate solution. The mixture was extruded dropwise into the CaCl₂ solution, as described in the preparation of BAMs. The resulting microspheres were stored at 4 °C. All procedures were conducted under sterile conditions. Field emission scanning electron microscopy (JEOL, JSM-IT710HR/JSM-IT210) was used to examine the microstructures of the BLBAMs. The diameter of the beads measured using an vernier caliper was approximately 2.5 mm. To confirm bacterial adherence to the biochar surface before alginate encapsulation, a portion of the biochar-bacteria mixture was air-dried and examined under scanning electron microscopy (SEM). The SEM micrographs revealed the presence of bacterial cells attached to the porous surface and within the cavities of the biochar, verifying successful colonization before immobilization.

### Biosorption experiments

Phenol biosorption experiments were carried out in a minimal salt medium (MSM) with pH 7, at 35 °C, for 48 h. A comparative examination was conducted between BAMs and BLBAMs as biosorbents for phenol biosorption. The absorbent dosage was standardized at 0.6 g/L. Biosorption trials were conducted using varying phenol concentrations in MSM: 25, 50, and 100 mg/L. The absorbents (BAMs and BLBAMs) were added separately and incubated with continuous shaking at 150 rpm. Control experiments, where no absorbent was added, were also performed.

### Phenol quantification and removal efficiency

The phenol Concentrations were measured using the Agilent 1100 High-Performance Liquid Chromatography (HPLC) system. Peaks were detected at 274 nm, eluting at 2.35 min using a C18 column, with a mobile phase consisting of a 20:80 mixture of phosphate buffer and acetonitrile, at a flow rate of 1.5 mL/min. The percentage of removal was determined according to the following equation:

1 $$\:R\% \: = \frac{{A_{i} - A_{f} }}{{A_{i} }}\:\:^{o} \:100$$

Where *R* was the removal percentage. The *A*_*1*_ and *A*_*f*_ were initial and final concentrations, respectively. All experiments were performed in triplicate to ensure precision. A bacteria-free medium served as a negative control. The data were subjected to statistical analysis via one-way ANOVA, with a significance level set at *P* < 0.05 to assess differences between the BAMs and BLBAMs methodologies regarding phenol removal efficiency.

### Characterization of BLBAMs

Various analytical techniques were employed to assess the structural and chemical properties of the BLBAMs. Scanning Electron Microscopy (SEM) (JEOL, JSM-IT710HR/JSM-IT210) was used to examine the biochar’s surface morphology and porosity. FTIR determined the functional groups of BLBAMs before and after phenol biosorption. The analysis was conducted with a Bruker Vertex 70 spectrophotometer (Billerica, MA, USA), configured for ATR measurements. The spectrum was obtained by scanning in the range of 400 to 4000 /cm. The surface area was calculated using the Brunauer–Emmett–Teller (BET) method through volumetric methods (a Micromeritics ASAP 2010) by adsorbing liquefied nitrogen at − 196 °C to generate isotherms, while the pore volume was derived from the nitrogen gas adsorbed at a (P/P_0_) value of 0.99.

### Optimization of phenol removal

The selected significant technique with the highest removal efficiency of BLBAMs was used to identify the optimal values of variables for attaining the best removal. The Response Surface Methodology (RSM), a commonly used statistical approach, was employed for optimizing variables to analyze different factors.

#### Experimental design

The RSM designed experiments, examined interactions among independent variables, adjusted these variables, and achieved optimal results. Each variable underwent testing at three levels: low, central, and high, as described in Table 1.

**Table 1:** Independent variables: coded and actual levels.

Variables	Coded and actual levels	Coded and actual levels		
		-1	0	+ 1
pH	A	5	6	7
Time (h)	B	18	24	30
Phenol concentration (mg/L)	C	25	50	75
Absorbent dose (g/L)	D	0.2	0.4	0.6

Equation (2) was utilized for coding the independent variables:

2 $$x_{i} ~~ = \frac{{X_{i} - X_{o} }}{X}$$

In this formula, *x*_*i*_ denoted the coded value of the test variable, *X*_*i*_ represented the uncoded value of the test variable, and *X*_*o*_ indicated the uncoded value of the variable at the central point. The Box-Behnken design (BBD) within the Response Surface Methodology (RSM) framework involved 27 tests to assess the combined effects of four independent factors on biosorption^23^.

The removal percentages measured experimentally were considered as the response. Equation (3) presented the mathematical relationship between the dependent and independent variables.

3 $$Y = ~\beta _{o} ~ + \mathop \sum \limits_{i} \beta _{i} X_{i} + ~\mathop \sum \limits_{{ii}} \beta _{{ii}} ~X_{i}^{2} + ~\mathop \sum \limits_{{ij}} \beta _{{ij}} ~X_{i} X_{j}$$

In this equation, *Y* represented the anticipated percentage of decolorization. The variables X_i_ and X_j_ were the uncoded independent variables. The term *β*_0_​ referred to the regression coefficient, *β*_*i*​_ signified the linear coefficient, *β*_*ii*​_ stood for the quadratic coefficient, and β_ij_​ denoted the interaction coefficient. Additionally, the positive signs in the equation suggested a synergistic effect of the factors, while the negative signs indicated an antagonistic effect.

#### Analysis of variance (ANOVA)

The response was predicted using a second-order polynomial equation derived from the Box-Behnken design, and regression analysis was conducted using an analysis of variance (ANOVA) with a 95% confidence level. The model’s fit was assessed using the determination coefficient R^2^ and adjusted R^2^. The contour plots were created to illustrate the interactions between the four independent variables and to confirm the optimization of variables that resulted from solving the second-order polynomial Eq. ^24^.

### Adsorption kinetics and isotherm

The phenol adsorption characteristics of BLBAMs were assessed using kinetic modeling approaches. In this study, pseudo-first-order and pseudo-second-order models were utilized to determine the best-fitting model for the observed data. Experiments were carried out in 250 mL Erlenmeyer flasks, each containing 200 mL of phenol solution and 0.2 g of BLBAMs. The experiments were conducted at a temperature of 35 °C, with a rotational speed of 150 rpm, and samples were collected at regular intervals of 18, 24, 30, and 36 h. Residual phenol concentration was measured by HPLC, and the quantity (*q*_*t*_) of adsorbed phenol per mass of microspheres (mg/g) was determined by Eq. (4).

4 $$q_{t} ~~ = \frac{{\left( {C_{o} ~{-}~C_{t} } \right)~~^{o} ~V}}{m}$$

Where *C*_*0*_ and *C*_*t*_ represented the initial and final concentrations (mg/L) of phenol, respectively, *V* was the volume of the solution, and *m* was the weight used for BLBAMs (g). Pseudo-first-order and pseudo-second-order models were employed to analyze adsorption kinetics following Eqs. (5) and (6).

5 $$Log10~\left( {q_{e} ~ - ~q_{t} } \right) = ~\log 10~q_{e} ~ - \frac{{k_{1} }}{{2.303}}~^{o} t~$$

6 $$\frac{1}{{q_{t} }} = \frac{1}{{K_{2} ~^{o} q_{e}^{2} }}~ + \frac{t}{{q_{e} }}$$

While *q*_*e*_ and *q*_*t*_ (mg/g) represented the adsorption capacities at equilibrium and at a specific time, respectively. *K*_*1*_ and *K*_*2*_ denoted the rate constants for the pseudo-first-order and pseudo-second-order models, respectively. Phenol solutions with concentrations ranging from 25 to 150 mg/L were prepared in separate conical flasks, each containing the same amount of BLBAMs (0.2 g/L dry weight) for the adsorption isotherm study.

The Langmuir, Freundlich, Sips, and Toth models were used to determine phenol adsorption isotherms following Eq. (7) to (10), respectively.

7 $$\frac{{C_{e} }}{{q_{e} }} = \frac{1}{{q_{{\max }} ~}}~^{o} C_{e} ~ + \frac{1}{{K_{L} ~^{o} q_{{\max }} ~}}~$$

8 $$\ln q_{e} = \ln K_{F} ~ + \frac{1}{n}\ln C_{e}$$

9 $$\frac{Q}{{q_{m} }} = \frac{{\left( {K_{P} ~C_{e} } \right)n}}{{1 + \left( {K_{{P~}} C_{e} } \right)n}}~^{o}$$

10 $$Q = Q_{{m~}} \left( {\frac{{\left( {K_{t} ~C_{e} } \right)}}{{1 + \left( {K_{{t~}} C_{e} } \right){\raise0.7ex\hbox{$1$} \!\mathord{\left/ {\vphantom {1 n}}\right.\kern-\nulldelimiterspace} \!\lower0.7ex\hbox{$n$}}}}~^{o} } \right)n~$$

Where *q*_*e*_ was the amount of adsorbate adsorbed per unit mass of adsorbent at equilibrium (mg/g), *C*_*e*_ represented the equilibrium concentration of the adsorbate (mg/L), *K*_*L*_ is the Langmuir adsorption constant, *q*_*max*_ is the maximum adsorption capacity (mg/g), *K*_*F*_ is the Freundlich constant, *1/n* is the constant related to the surface heterogeneity, and *K*_*P*_ and K_t_ are the Sips and Toth constant, respectively.

### Reusability study of BLBAMs

To determine the reusability of BLBAMs, the microspheres were reused in multiple experiments, and their phenol removal efficiency was evaluated after each cycle. After each adsorption cycle, BLBAMs were recovered by filtration and regenerated by shaking in 0.1 M NaOH solution for 1 h to desorb bound phenol, followed by thorough rinsing with sterile distilled water to remove residual alkali. The beads were then neutralized using phosphate buffer (pH 7) and reused under the same optimized phenol removal conditions. This regeneration protocol was repeated for four consecutive cycles to assess the reusability of the biosorbent.

#### Statistical analyses

Data management was performed using Microsoft Office Excel (2016), while Minitab 18 facilitated experimental design, data analysis, and interpretation. A one-way ANOVA was employed to analyze the data, with statistical significance determined at a threshold of *P* < 0.05, to assess differences in phenol removal efficiency between BAMs and BLBAMs methodologies. Additionally, ANOVA was employed to evaluate the influence of various factors.

## Results and discussion

After isolating and screening multiple bacterial strains, the strain that demonstrated the highest efficiency in removing more than 80% of phenol at an initial concentration of 100 mg/L was selected for further experimentation. To identify this isolate, 16 S rDNA sequencing technology was used, and the sequence was compared with reference genes in GenBank. The analysis revealed that the isolate is *Salinivibrio kushneri*. Its gene sequence has been officially submitted to the NCBI GenBank and is cataloged under the unique accession number PQ836117. The specific isolate (*Salinivibrio kushneri* PQ836117) exhibited superior performance relative to others, achieving a phenol removal rate exceeding 80% at pH 7 and 35 °C after 48 h, with a phenol concentration of 100 mg/L. Elevated phenol concentrations can significantly inhibit or damage bacterial strains, reducing their efficacy in managing high phenol levels.

Statistical analysis using one-way ANOVA revealed that phenol removal by BLBAMs was significantly higher than that by BAMs (*P* = 0.035), confirming the enhanced performance due to biochar integration. This improvement is attributed to biochar’s porous structure and functional surface chemistry, which enhance microbial colonization, protect cells from toxic compounds, and facilitate nutrient retention ^25,10^. Biochar also promotes biofilm development and supports microbial survival under environmental stress, improving overall biodegradation performance^2,9^.

### Characterization of BLBAMs

**Fig. 1:**
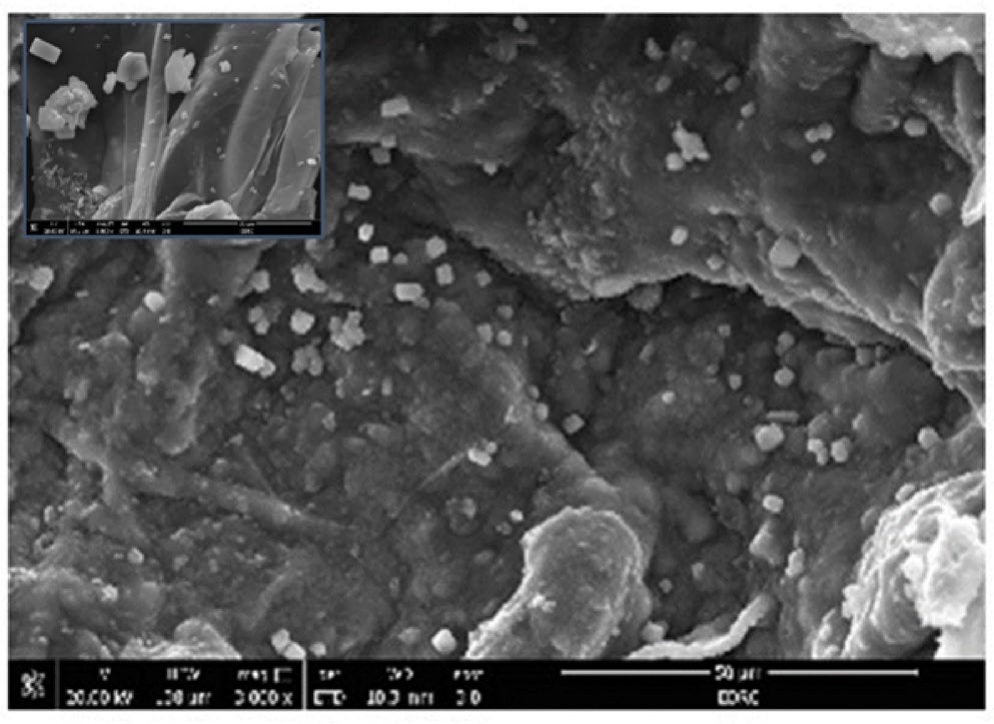
SEM micrographs of BLBAMs showing porous structure and bacterial immobilization.

**Fig. 2:**
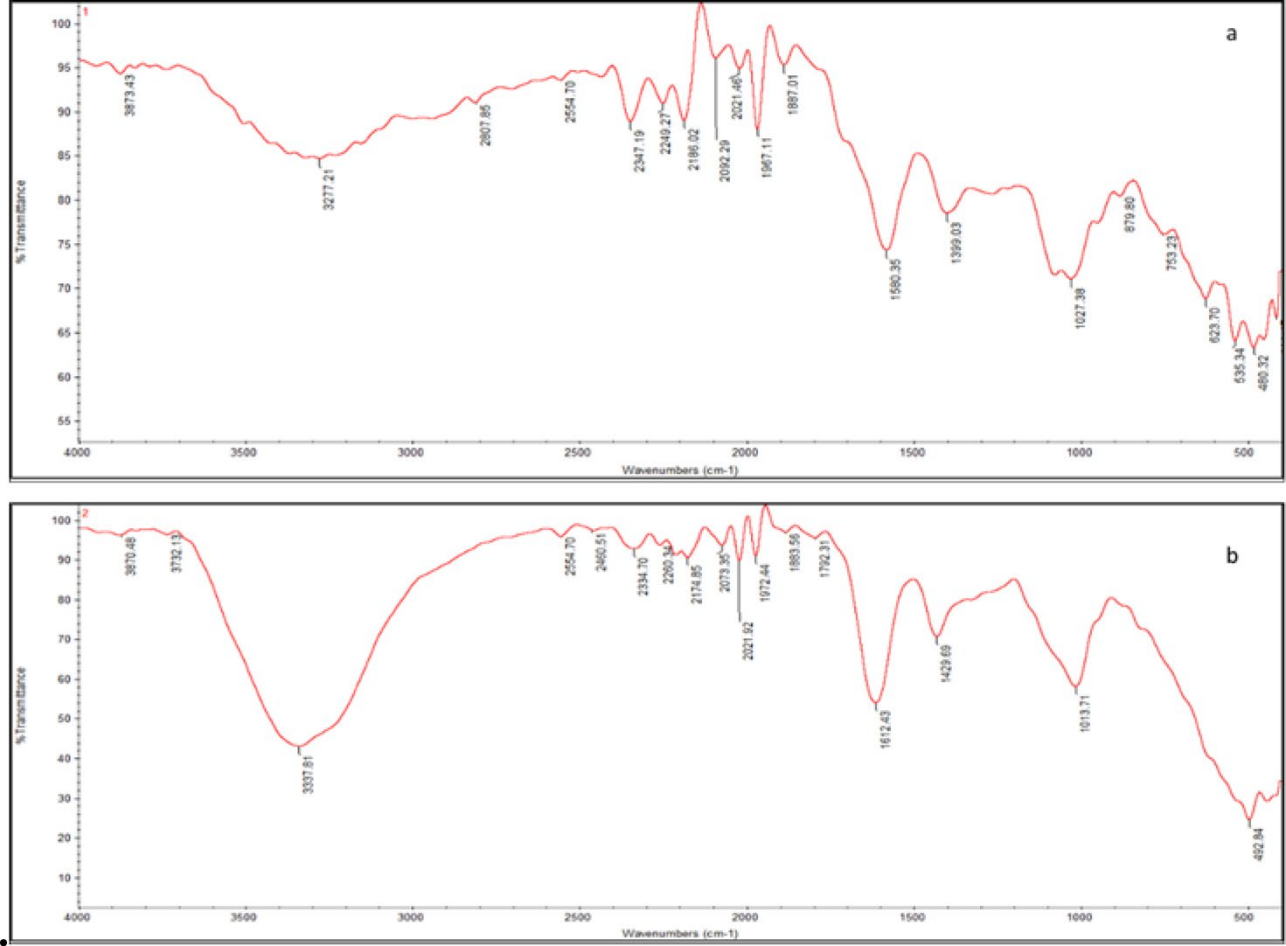
The FTIR spectrums of BLBAMs before (**a**) and after (**b**) phenol biosorption.

The biochar derived from water hyacinth exhibited a highly porous structure, as confirmed by Scanning Electron Microscopy (SEM), which enhances adsorption capacity. The SEM images (Fig. 1) illustrated that the irregular surface morphology of biochar and porous structure facilitated bacterial adherence. Numerous bacterial cells appeared embedded and scattered across the pore spaces. Fig.S1(Supplementary material) displayed the N_2_ adsorption and desorption isotherm curves for BLBAMs. Upon conducting (BET) analysis, it was observed that BLBAMs exhibited a type IV adsorption isotherm^6^. According to the IUPAC classification, the adsorption isotherms of these materials aligned with H3-type hysteresis loops, the adsorption behavior may plateau or exhibit hysteresis due to capillary condensation, suggesting the presence of well-defined slit-like mesoporous filling mechanism within the structures^5^. The BET results, including the specific surface area (SBET​= 7.1965m^2^/gm²/g), total pore volume (VT​​=0.07876cm^3^/g), and mean pore radius (*r* = 4.2011 nm) of the solid adsorbents. The FTIR (Fig. 2) was performed on BLBAMs before and after biosorption to analyze any change in functional groups due to interaction with phenol. The FTIR spectra confirmed the presence of hydroxyl (OH), carbonyl (C = O), and aromatic functional groups, all of which play a crucial role in phenol binding (Fig. 2a). The FTIR spectra exhibited significant changes in the functional groups before and after biosorption (Fig. 2a and b). The broad absorption peak observed around 3200–3600/cm for hydroxyl functional groups^4^ weakened after biosorption, indicating the active involvement of their hydroxyl groups in hydrogen bonding with phenol molecules. The aliphatic and aromatic C-H stretching bands in the 2800–3000/cm range showed a reduction of intensity, reflecting interactions with the phenol structure^10^. The carbonyl stretching band has been shifted to 1650–1750/cm for hydrogen bonding or other types of interaction^26^. The peaks of the C = C stretching of aromatic rings in the region of 1400–1600/cm increased in intensity after phenol biosorption onto the surface of BLBAMs, thus providing evidence for the presence of the aromatic ring typical of phenol on the surface of BLBAMs^15^. Furthermore, phosphate (P = O) groups in the spectral range of 1050–1250/cm revealed small shifts, thus supporting their involvement in the process of adsorption^27^. These results confirmed the contribution of functional groups like hydroxyl, carbonyl, and aromatic rings in phenol biosorption, demonstrating the BLBAMs’ efficiency.

### Optimization of the phenol removal process

The BLBAMs were further investigated to identify the best conditions for achieving maximum removal. To better understand how different environmental factors interact, the BBD was implemented, following the approach of ^28^. The study focused on four key factors: pH, interaction duration, phenol concentration, and biosorbent amount. The experimental data were processed using RSM, which helped develop an empirical model to predict the optimal conditions for phenol removal. Twenty-seven experiments were conducted using BBD. The results for both the experimental and predicted phenol biosorption are shown in Table 2.

**Table 2:** Twenty-seven experimental and predicted results of the BBD.

Run No.	A	B	C	D	Removal (%)	
					Actual	Predicted
1	-1	0	-1	0	20.77	19.4525
2	0	1	-1	0	48.27	51.2354
3	0	0	-1	-1	46.09	45.0138
4	0	0	0	0	52.13	53.8333
5	0	0	1	1	48.03	50.0021
6	-1	1	0	0	30.98	33.5454
7	0	1	0	-1	53.11	50.5642
8	1	0	0	1	78.19	79.3971
9	0	1	0	1	69.70	66.3358
10	-1	-1	0	0	16.65	13.6387
11	-1	0	1	0	21.36	21.8742
12	0	0	1	-1	37.33	40.9154
13	0	0	-1	1	55.23	52.5404
14	0	-1	-1	0	40.11	44.2538
15	1	-1	0	0	66.09	64.4204
16	0	-1	1	0	33.46	31.1104
17	0	-1	0	-1	39.37	41.2225
18	0	0	0	0	55.07	53.8333
19	-1	0	0	1	24.00	25.8404
20	0	-1	0	1	41.03	42.0642
21	0	0	0	0	54.30	53.8333
22	1	0	-1	0	74.90	72.8742
23	0	1	1	0	61.27	57.7421
24	1	0	0	-1	66.44	65.2154
25	-1	0	0	-1	24.00	23.4087
26	1	0	1	0	64.01	63.8158
27	1	31	0	0	74.22	78.1271

The BBD created the subsequent quadratic Eq. (11) to predict the phenol biosorption % primarily based on the variables that have been evaluated.

11 $$\begin{aligned} Y~ = & ~ - 302.6~ + ~77.8~A~ + ~3.28~B~ + ~0.659~C~ - ~118.4~D~ - ~3.99~A^{2} ~ - ~0.0669~B^{2} ~ \\ & - ~0.00854~C^{2} ~ - ~34.4~D^{2} ~~ - ~0.258~A~^{o} B~ - ~0.1148~A~~^{o} C~ + ~14.69~A~~^{o} D~ \\ & + ~0.0328~B~~^{o} C~ + ~3.11~B~~^{o} D~ + ~0.078~C~~^{o} D~ \\ \end{aligned}$$

**Fig. 3:**
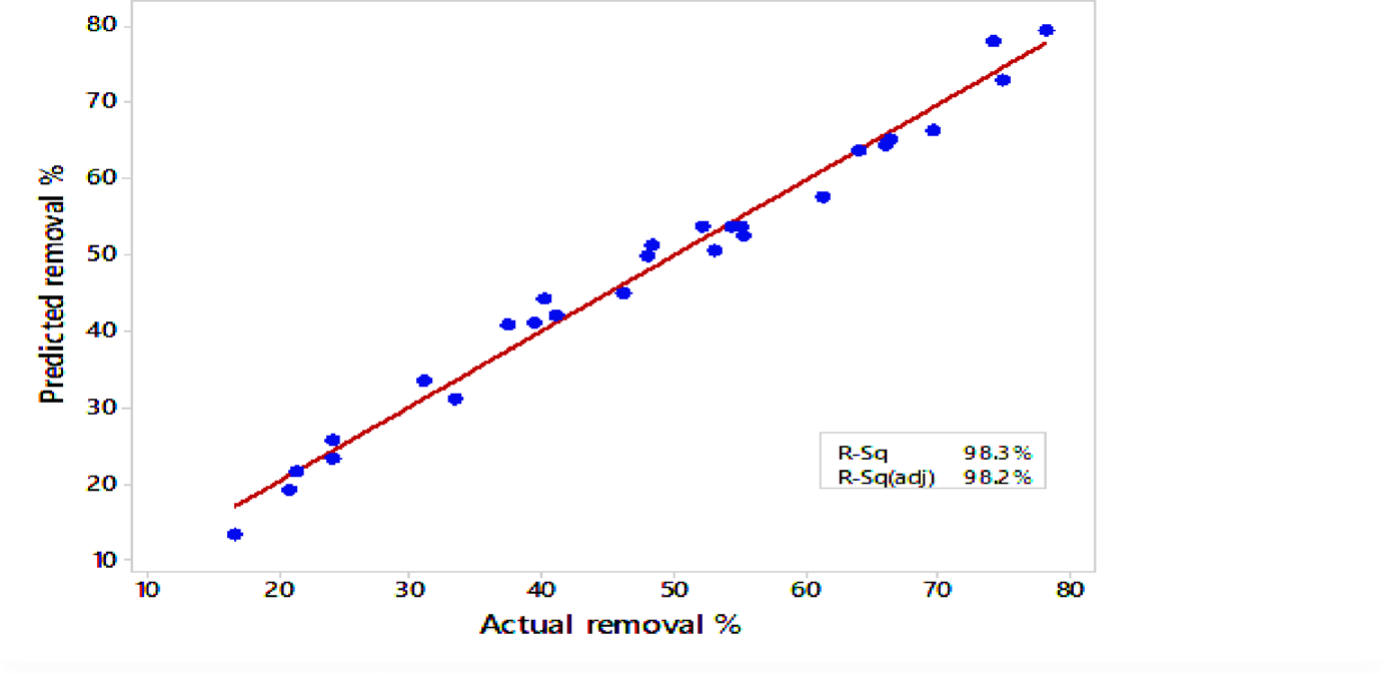
Correlation between observed and predicted phenol removal efficiency (%) using the response surface methodology (RSM) model.

Where *Y* represented the anticipated removal and variables were *A*, *B*, *C*, and *D*, which stood for pH level, interaction duration, phenol concentration, and dose, respectively. The analysis of variance (ANOVA) was used to evaluate the validity of the design model at a 95% confidence level. The *F*-value and the *P*-value were used to assess the model. The model’s *P*-value (0.000) indicated a statistically significant relationship in predicting the elimination percentage using BLBAMs. The results (Table 3) showed that the interactions, square, and linear effects were all statistically significant (*P*-value < 0.05). The model proved to be valid and significant in explaining the process, as evidenced by the non-significant lack of fit (*P*-value = 0.153). The high *R*^*2*^ value (Fig. 3) showed a good correlation between the observed and predicted responses.

**Table 3:** ANOVA for phenol removal percentage by BLBAMs.

Source	Degrees of freedom	Sum of square	Mean square	F-Value	*P*-Value
Model	14	8325.64	594.69	50.06	0.000
Linear	4	7908.05	1977.01	166.42	0.000
pH	1	6820.62	6820.62	574.15	0.000
Time	1	847.39	847.39	71.33	0.000
Concentration	1	33.03	33.03	2.78	*0.121*
Dose	1	207.00	207.00	17.43	0.001
Square	4	187.65	46.91	3.95	0.029
pH*pH	1	84.94	84.94	7.15	0.020
Time*Time	1	30.97	30.97	2.61	*0.132*
Concentration*Concentration	1	151.99	151.99	12.79	0.004
Dose*Dose	1	10.11	10.11	0.85	*0.374*
Interaction	6	229.94	38.32	3.23	0.040
pH*Time	1	9.61	9.61	0.81	*0.386*
pH*Concentration	1	32.95	32.95	2.77	*0.122*
pH*Dose	1	34.52	34.52	2.91	*0.114*
Time*Concentration	1	96.53	96.53	8.13	0.015
Time*Dose	1	55.73	55.73	4.69	*0.051*
Concentration *Dose	1	0.61	0.61	0.05	*0.825*
Error	12	142.55	11.88		
Lack-of-fit	10	137.90	13.79	5.93	0.153
Pure error	2	4.65	2.32		
Total	26	8468.19			
R^2^		0.9832			
Adjusted R^2^		0.9635			
Predicted R^2^		0.9050			

**Fig. 4:**
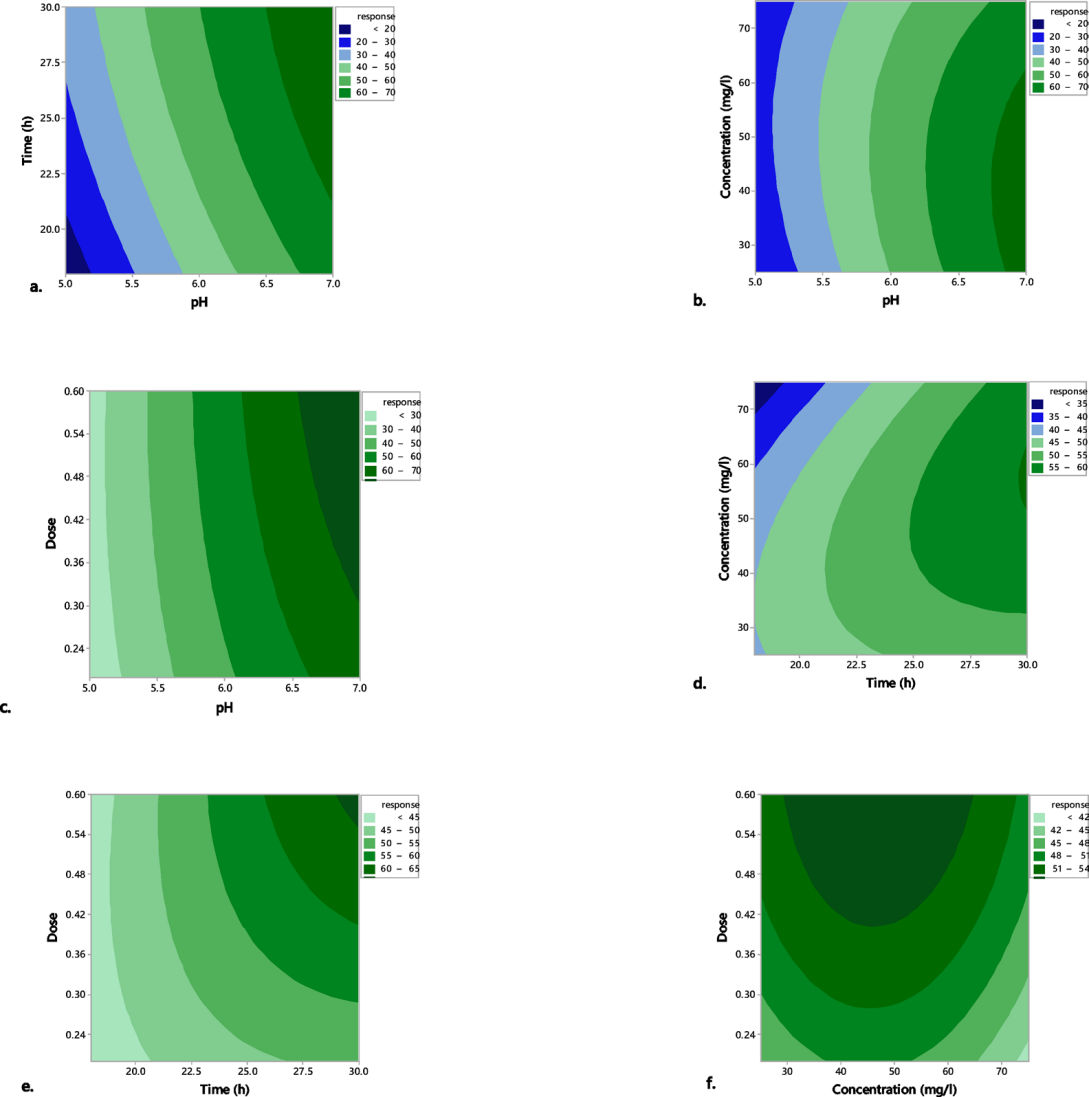
Contour plots of phenol removal efficiency% (response) and two independent variables.

The *R*^*2*^ indicated that 98.3% of the variances in the removal process could be explained by the model. The adjusted *R*^*2*^ and predicted *R*^*2*^ values supported the importance of the model. An Adjusted *R*^*2*^ of 96.35%, close to the *R*^*2*^, showed a strong correlation between the response and the model. Moreover, the predicted *R*^*2*^ of 90.50% indicated that the model could reliably predict the response. The contour plots (Fig. 4) were made by mapping the removal percentage (response) against two independent variables while holding the remaining variables at their central values (coded as 0).

Contour plots were created to show the correlations between the variables and to verify the ideal values of the variables discovered through the solution of the second-order polynomial equation. The interaction effect between pH and contact time was illustrated in Fig. 4a. It was observed that biosorption efficiency increased with a rise in pH up to 7 and a contact time of 30 h, aligning with the optimal values determined by the equation. The optimal biosorption observed at pH 7 is primarily due to the chemical properties of both phenol and the biosorbent under these conditions. At pH 7, phenol remains mostly uncharged (as its pKa is approximately 9.9), while the biosorbent surface, enriched with deprotonated carboxyl and hydroxyl groups, carries a net negative charge. Although electrostatic interactions are minimal due to phenol’s neutral state, biosorption is enhanced through hydrogen bonding, π–π stacking between aromatic rings, and structural stabilization of the BLBAMs. Additionally, a pH of 7 promotes high microbial activity, which further enhances phenol removal efficiency. The neutral pH also helps preserve the structural integrity of both the bacterial cells and the immobilization matrix, which is essential for sustained and effective biosorption^29^. As shown in Fig. 4a, d, and e, the contact time influenced the kinetics and the overall efficiency of phenol removal. In the initial phase, the biosorption occurred quickly due to the presence of a significant number of binding sites. As the initial binding sites became occupied, the biosorption rate started to slow down to the equilibrium level. The biosorption process has reached its maximum capacity, and the immobilized bacteria cannot adsorb additional phenol molecules^30^. Phenol concentration was another critical factor in the biosorption process. Increasing the phenol concentration in the solution led to biosorption sites becoming more saturated quickly (Fig. 4d), leading to a plateau in the biosorption capacity once equilibrium was reached^31^. It was evident from Fig. 4c, e, and f that a high biosorbent dose increased available binding sites for phenol molecules. This generally enhanced the biosorption capacity of the system. More biosorbents provided more surface area and active sites for interaction with phenol^25^.

**Fig. 5:**
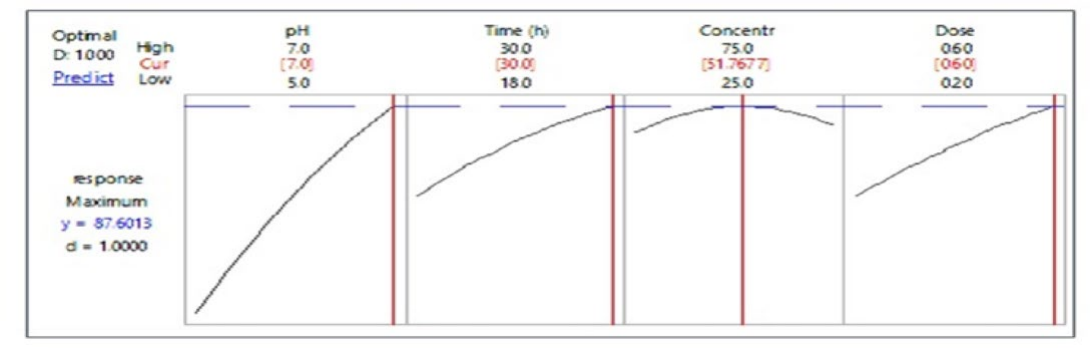
Optimization plot generated using Minitab 18 showing predicted maximum phenol removal at optimal levels of pH, contact time, phenol concentration, and BLBAM dose.

After solving the second-order polynomial equation using the optimizer in Minitab 18, and analysis of Fig. 5, the maximum predicted phenol biosorption of 87.60 was recommended at the optimum predicted pH 7, interaction duration of 30 h, phenol concentration of 51.76 mg/L, and absorbent dose of 0.6 g/L.

The enhanced removal efficiency is due to the porous structure and functional surface chemistry of biochar, which increases the availability of binding sites, encourages bacterial adherence, and shields microbial cells from toxic stress. Similar results have been shown in previous studies^30^. reported phenol removal efficiencies of over 80% using calcium alginate biochar microspheres with immobilized *Bacillus sp*., highlighting the benefit of biochar addition compared to alginate alone. ^32^ observed phenol removal efficiencies exceeding 85% with biochar-immobilized *Serratia sp*. Another study by ^33^ found that *Pseudomonas citronellellolis* immobilized on peanut shell-derived biochar achieved high phenol removal rates, with 99.2% at 100 mg/L and 82% at 200 mg/L. ^10^ noted phenol removal of 85–90% using a biochar–bacteria system, emphasizing how porous biochar supports microbial stability and adsorption. Similarly,^6^ achieved 83–86% phenol removal with a biochar–Delonix regia seed gum chitosan composite, attributing the enhancement to the presence of mesoporosity and functional groups. In comparison, the BLBAMs created in this study not only match but slightly surpass these efficiencies at 87.6%, making them among the most effective biochar-based systems reported so far.

### Kinetic and isotherm studies

Two kinetic models to explore the adsorption kinetics mechanism of phenol onto BLBAMs were used: PFO (Eq. 5), and PSO (Eq. 6). The PFO is used for the physisorption process, and the adsorption rate is directly linked to the availability of adsorption sites. The PSO is indicated in the chemisorption process, and the adsorption rate is directly linked to the square of the number of adsorption sites^9,34^. The models were evaluated based on the linearity of the fit (*R²*) ^35^ and the consistency between the experimental and predicted values of *q*_*e*_ (Table 4). Adsorption of phenol was monitored at various time intervals.

**Table 4:** Kinetic parameters for pseudo-first order and pseudo-second order models.

Model	Kinetic parameter	Calculated value
Pseudo-first-order (PFO)	*K*_*1*_ (1/min)	0.0029
	*q*_*e*_ (mg/g)	40.869
	*R* ^*2*^	0.885
Pseudo-second-order (PSO)	*K*_*2*_ (g/(mg/min))	0.00019
	*q*_*e*_ (mg/g)	26.455
	*R* ^*2*^	0.999

**Fig. 6:**
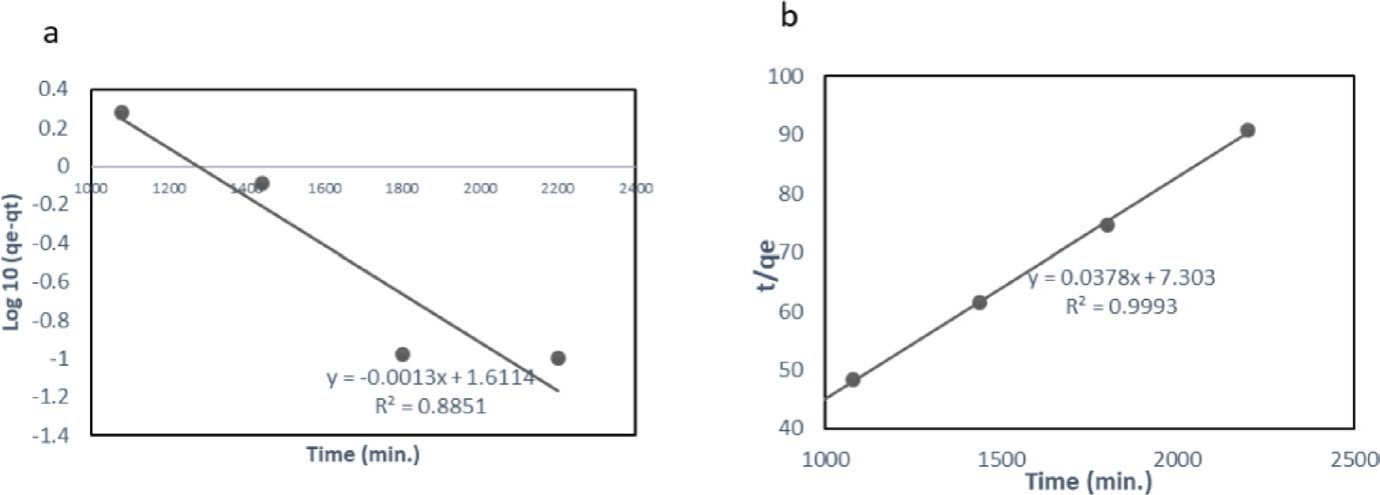
Phenol adsorption kinetics onto BLBAMs: (a) pseudo-first-order model and (b) pseudo-second-order model.

For the PFO model, values of *K*_*1*_ (0.0029), *q*_*e*_ (40.869), and *R*^*2*^ (0.885) were obtained for the BLBAMs. Based on the PSO kinetic model, phenol adsorption onto BLBAMs demonstrated a *K*_*2*_ value of 0.00019 and a *q*_*e*_ value of 26.455. The observed *R*^*2*^ value for phenol was 0.999. These values (Fig. 6) indicated that the adsorption of phenol on BLBAMs followed the PSO model. The *R*^*2*^ of PSO was significantly higher than PFO, and the *qe* of PSO matched the experimental data.

**Fig. 7:**
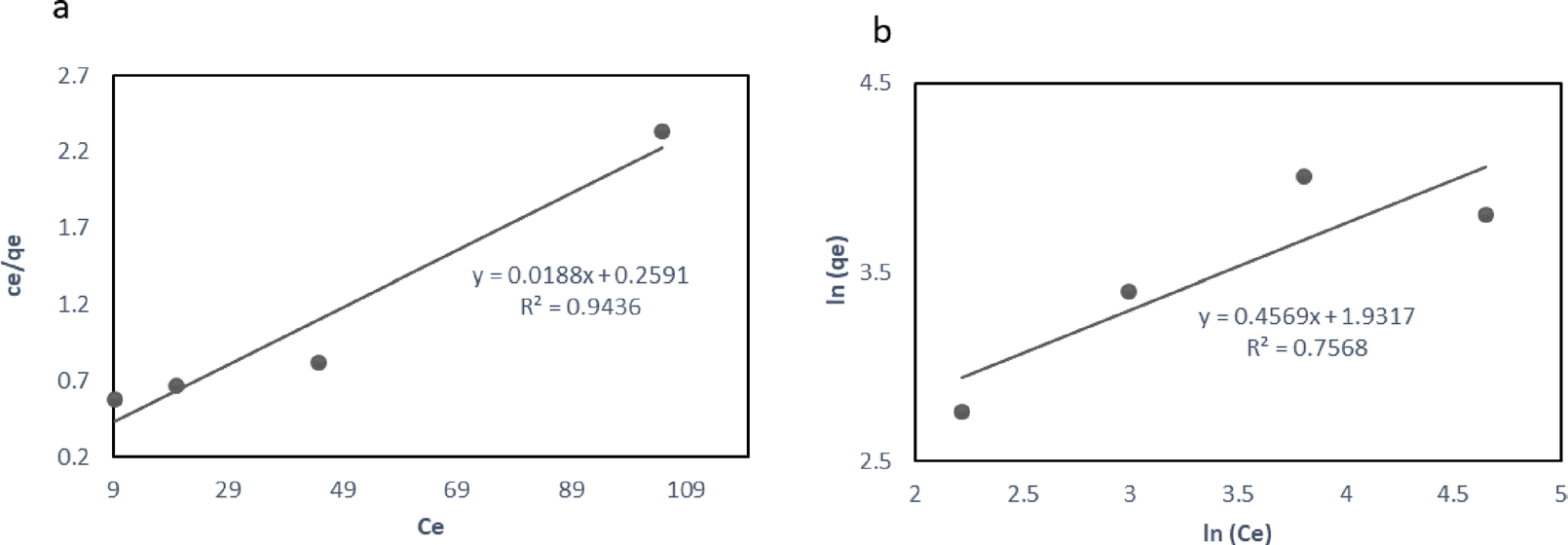
Adsorption isotherms for phenol uptake by BLBAMs fitted with (**a**) Langmuir and (**b**) Freundlich models.

Isotherm equations describe the behavior and capacity of the adsorbent. The Langmuir model describes monolayer adsorption occurring on a uniform surface, whereas the Freundlich model accounts for multilayer adsorption on a non-uniform, heterogeneous surface^34^. The data fit into Langmuir or Freundlich refers to the process nature. The *R*^2^ of Langmuir, Freundlich’s, Sips, and Toth models evaluate the degree to which the mathematical model corresponds to the experimental data^34,36^. Both empirical models, the Sips and the Toth isotherms, explain how chemicals adsorb onto surfaces, especially when heterogeneity is present. According to the Sips model results, the fitted parameters show an empirical parameter (n_s_) of 2, a maximum adsorption capacity (*q*_mcal_) of 50 mg/g, and a Sips constant (*K*_s_) of 0.02 L/mg. According to these parameters, the adsorption process exhibits a sigmoidal pattern, whereby the affinity increases with increasing concentration until it reaches saturation. The Toth isotherm, on the other hand, incorporates an extra parameter (t) to account for surface heterogeneity, offering a more comprehensive explanation of adsorption. The same dataset was also subjected to the Toth model, which produced a heterogeneity factor (n_t_) of 1.8, a Toth constant (*K*_t_) of 0.012 L/mg, and a qm_cal_ value of 58 mg/g. Because the Toth model is better at capturing heterogeneous surface interactions, its higher q m value suggests a marginally larger anticipated adsorption capacity than the Sips model. Both models showed relatively high *R*² values (0.92–0.94), indicating that they were appropriate for explaining the adsorption process. However, the Toth model is especially useful when working with complex systems or materials that have non-uniform pore distributions because of its adaptability to different levels of heterogeneity. The investigation shows that the Toth model offers a more sophisticated knowledge of heterogeneous adsorption processes, whereas the Sips model is excellent at fitting homogeneous adsorption processes. Table S1and Fig. 7 showed a stronger alignment of the adsorption data with the Langmuir and Sips models (*R*^*2*^ = 0.943 and 0.92), respectively, compared to the Freundlich model (*R*^*2*^ = 0.756). This model inferred that phenol adsorption by BLBAMs occurred in monolayers and homogeneous sites.

The discrepancy between the PSO-derived q_e_ (26.455 mg/g) and the Langmuir q_max_ (53.191 mg/g) is attributed to differing experimental scopes. The PSO model reflects the adsorbed amount under a single concentration and specific contact time. The Langmuir model estimates a theoretical maximum based on equilibrium adsorption data across a range of concentrations. Thus, although the PSO model fit the kinetic data very well (*R*² = 0.999), it did not reach the maximum adsorption capacity observed in the isotherm study.

### Adsorption mechanism using BLBAMs

The pseudo-second-order kinetic model (*R*^2^ = 0.999) confirmed that chemisorption was the primary mechanism governing phenol adsorption onto BLBAMs. A crucial aspect of this process was the interaction between phenol molecules and the functional groups present in BLBAMs. FTIR analysis revealed the involvement of aromatic, carbonyl (C = O), and hydroxyl (-OH) groups in phenol binding. Adsorption occurred through multiple mechanisms: π–π interactions facilitated electron cloud interactions between the aromatic rings of biochar and phenol, while hydrogen bonding took place between the hydroxyl groups of BLBAMs and phenol. This mechanism was further supported by the increased intensity of C = C stretching bands in the 1400–1600 cm⁻¹ range after adsorption. Electrostatic interactions at an optimal pH of 7 contributed to maintaining the biosorbent’s structural integrity and charge balance. Additionally, van der Waals forces enhanced adsorption by strengthening physical attractions between phenol molecules and the micropores of BLBAMs. Changes in the carbonyl stretching bands (1650–1750 cm⁻¹) suggested their involvement in adsorption, potentially through electron transfer or hydrogen bonding. The adsorption isotherm results, closely aligned with the Langmuir model (*R*² = 0.943), indicated monolayer adsorption on uniform active sites. These findings demonstrate that phenol adsorption onto BLBAMs was driven by multiple interactions, with chemisorption being the dominant process, explaining the high removal efficiency observed in this study.

The superior performance of BLBAMs compared to BAMs can be attributed to the synergistic integration of biochar’s physicochemical properties with microbial biodegradation. The pseudo-second-order kinetic model (R² = 0.999) confirmed that chemisorption governed the adsorption process, indicating strong interactions between phenol molecules and functional groups such as hydroxyl and carbonyl, as revealed by FTIR analysis. This finding aligns with previous studies that reported biochar–bacteria composites achieving high phenol removal efficiencies (e.g., Bacillus sp. immobilized on calcium alginate biochar at > 80%) but extends the understanding by demonstrating slightly higher efficiencies (87.6%) using water hyacinth-derived biochar, a sustainable and low-cost feedstock. Moreover, the close fit to the Langmuir isotherm (qmax = 53.19 mg/g, R² = 0.943) suggests that phenol binding predominantly occurred as monolayer adsorption on uniform active sites, reinforcing the hypothesis that biochar’s porous structure not only provides additional surface area but also stabilizes microbial colonization.

### Reusability study of BLBAMs

**Fig. 8:**
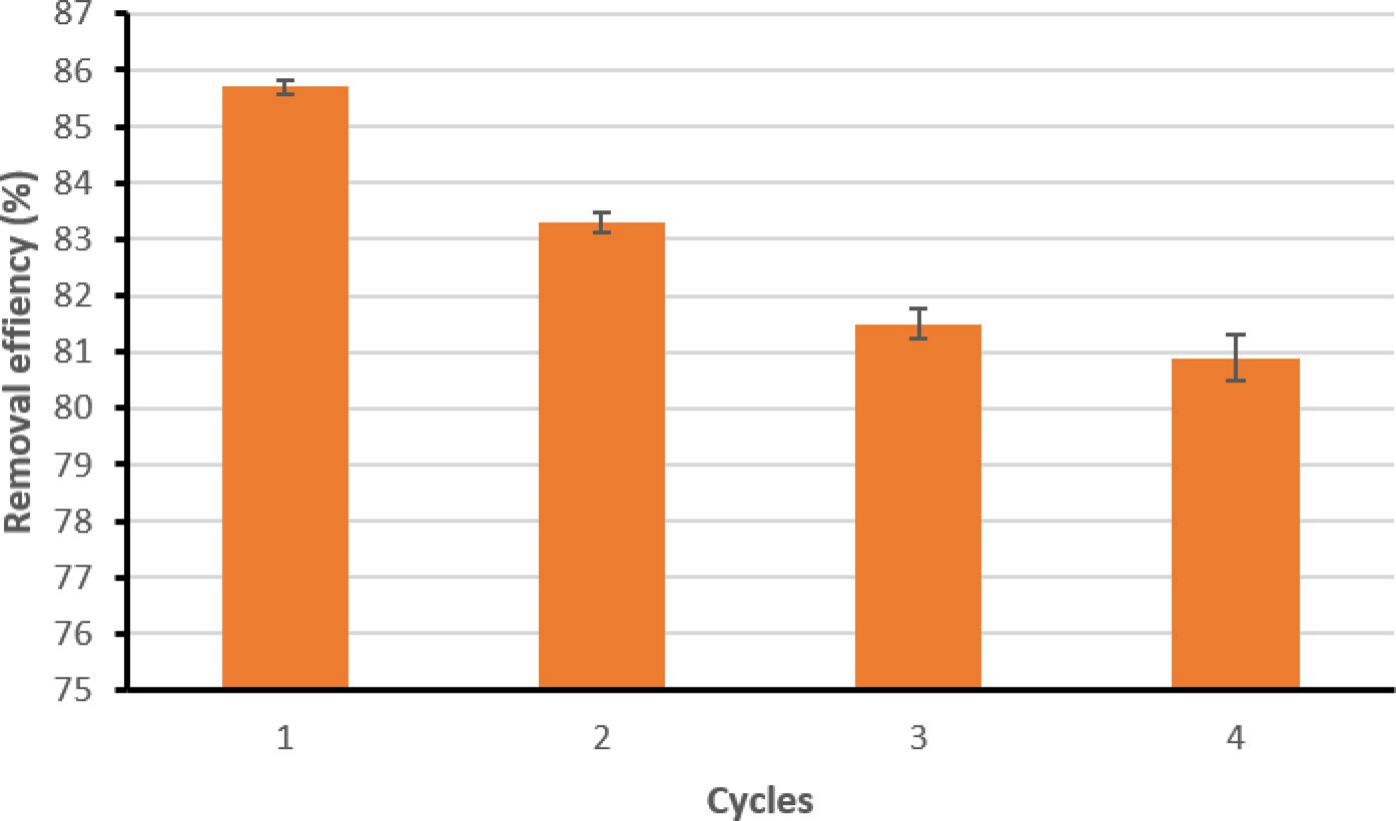
Reusability study of BLBAMs for phenol adsorption after various regeneration cycles.

Recycling the biosorbent is crucial for large-scale applications to reduce overall process costs and minimize the continuous demand for new raw materials. Four consecutive cycles were conducted to evaluate the phenol removal efficiency of BLBAMs. The initial efficiency in the first cycle was 83.51 ± 0.6%, which decreased slightly to 80.09 ± 0.8% after the fourth cycle, representing only a 4% reduction (Fig. 8). This modest decline may be attributed to several factors: (i) partial microbial leakage or reduced metabolic activity under repeated phenol exposure, which lowers biodegradation efficiency; (ii) minor structural degradation of the biochar–alginate matrix, such as relaxation of the alginate network or microfractures at the biochar–polymer interface, leading to decreased stability; and (iii) incomplete desorption of phenol and its intermediates, which could progressively block active sites. Similar gradual declines have been observed in other biochar-based immobilization systems^2,6^, where surface fouling and microbial stress were identified as limiting factors. Future studies could mitigate these issues by incorporating co-crosslinking agents to strengthen the matrix and reduce leakage, optimizing regeneration protocols to improve desorption, and employing periodic re-crosslinking or nutrient supplementation to sustain microbial activity. Despite this slight decline, the relatively stable performance of BLBAMs across cycles demonstrates their durability and strong potential for long-term application in wastewater treatment.

### Adsorption capacities of various adsorbents for phenol removal

Recent literature (2016–2024) has documented the maximum adsorption capacity (*q*_m_) of various adsorbents. In comparison with other reported adsorbents (Table 5) such as sugarcane bagasse (57.5 mg/g), coconut shell activated carbon (21.2 mg/g), and calcium alginate–MWCNT beads (58.8 mg/g), the maximum adsorption capacity of BLBAMs (53.19 mg/g) is among the highest values reported for sustainable and reusable biosorbents. This comparative positioning highlights the competitive advantage of BLBAMs within the broader field of phenol remediation, confirming their role as a highly effective, eco-friendly, and cost-efficient solution.

**Table 5:** Comparison of maximum adsorption capacities for phenol using various adsorbents.

Adsorbent	q_max_ (mg/g)	Removal (%)	Reusability (efficiency after reuse)	Reference
BLBAMs (this study)	53.19	87.6	80.09% after 4 cycles	This study
Sugarcane bagasse	57.5	85	‒	^12^
Pomelo peel	39.3	78	‒	^37^
Palm shell-based carbon	19.58	60	‒	^14^
Coconut shell activated carbon	21.21	66.3	‒	^13^
Graphene oxide–PNIPAM	12.7	58	47% after 3 cycles	^17^
Hevea brasiliensis seed shell biochar	13.3	70	‒	^15^
Phoenix dactylifera leaf	14.3	72	‒	^16^
Calcium alginate–MWCNT beads	58.8	90	71.2% after 3 cycles	^38^
Eucalyptus globulus seed biochar	27.3	80	‒	^39^

#### Limitations and future work

Despite promising results, this study has limitations, including enhancing BLBAM stability, preventing microbial leakage, and developing efficient regeneration methods for long-term use. Real-world applicability must be assessed under varying wastewater conditions, and large-scale biochar production requires economic evaluation. Additionally, integrating BLBAMs into existing treatment systems presents technical challenges. Addressing these factors will optimize the technology for practical use. Future research should assess BLBAM performance in real industrial wastewater settings, optimize biochar modification for improved adsorption, and develop advanced regeneration techniques for long-term use. Additionally, integrating BLBAMs into existing treatment systems should be explored to evaluate scalability and feasibility. Further studies on removing other persistent organic pollutants could expand their environmental applications.

## Conclusions

This study demonstrated the effectiveness of bacteria-loaded biochar–alginate microspheres (BLBAMs) for phenol removal. Under optimized conditions (pH 7, 30 h contact time, 51.76 mg/L phenol concentration, and 0.6 g/L biosorbent dose), BLBAMs achieved a removal efficiency of 87.6%. Phenol adsorption followed a pseudo-second-order kinetic model (R² = 0.999), indicating chemisorption, and fit the Langmuir isotherm (R² = 0.943), with a maximum capacity of 53.19 mg/g. The BLBAMs also demonstrated stability and reusability, retaining 80.09% efficiency after four cycles, confirming their long-term effectiveness. Importantly, the system—prepared from invasive water hyacinth and incorporating Salinivibrio kushneri—offers a novel, durable, and environmentally sustainable solution for wastewater treatment. By integrating alginate’s biocompatibility, biochar’s high porosity and functional groups, and the microbial degradation capacity of Salinivibrio, BLBAMs overcome key limitations of conventional immobilization methods such as weak mechanical stability, microbial leakage, and low reusability. Beyond their strong adsorption and biodegradation performance, the use of water hyacinth advances waste-to-resource valorization, aligning pollutant remediation with circular economy principles. Collectively, these findings highlight the contribution of BLBAMs to the advancement of bioremediation, offering a scalable, cost-effective, and sustainable strategy for the future of wastewater treatment.

## Supplementary Information

Below is the link to the electronic supplementary material.

Supplementary Material 1

## Data Availability

The datasets used and/or analysed during the current study available from the corresponding author on reasonable request.
