# Supplementary material for: Synergistic strategies of Salinivibrio kushneri immobilization on biochar alginate microspheres for the removal of phenol from aqueous solution modeling approaches
Source: Sci Rep. 2025 Oct 1;15:34289. doi: 10.1038/s41598-025-20677-4 (PMC12488877; doi:10.1038/s41598-025-20677-4)

***Supplementary Materials***

**Synergistic strategies of *salinivibrio kushneri* immobilization on biochar-alginate microspheres for the removal of phenol from aqueous solution: experimental and modeling approaches**

^1^ Mohammed T. M. H. Hamad and ^2^ Walaa S. Mohamed

^1^Microbiology Department, Central Laboratory for Environmental Quality Monitoring (CLEQM), National Water Research Center (NWRC), Cairo, Egypt.

E-mail: [mohamed_taha@nwrc.gov.eg](mailto:mohamed_taha@nwrc.gov.eg)

^2^Biology and Environmental Indicators Department, Central Laboratory for Environmental Quality Monitoring (CLEQM), National Water Research Center (NWRC), Cairo, Egypt.

E-mail: [walaa_mohamed@nwrc.gov.eg](mailto:walaa_mohamed@nwrc.gov.eg)

**Corresponding author:**

Mohammed T.M.H.Hamad

**E-mail:** [moham](mailto:mohammedtaha2010@outlook.com)ed_taha@nwrc.gov.eg

**Contents**

**Fig. S1 The N2 adsorption−desorption curves of** BLBAMs**.**

**Table S1** Isotherm parameters for Langmuir, Freundlich, Sips and Toth models.

**Table S1** Isotherm parameters for Langmuir, Freundlich ,Sips and Toth models.

| Model | Kinetic parameter | Calculated value |
| --- | --- | --- |
| Langmuir | *q_max_* (mg/g) | 53.191 |
|  | *K_L_* (L/mg) | 0.072 |
|  | *R^2^* | 0.943 |
| Freundlich | *n* | 2.188 |
|  | *K_F_* (mg/g) | 6.901 |
|  | *R^2^* | 0.756 |
| Sips | *q_max_* (mg/g) | 50.1 |
|  | n_s_ | 2 |
|  | *K*_s_ (L/mg) | 0.02 |
|  | *R^2^* | 0.92 |
|  |  |  |
| Toth | *q_max_* (mg/g)  *n_t_*  *K_t_* (L/mg)  *R^2^* | 58  1.8  0.012  0.902 |
|  |  |  |


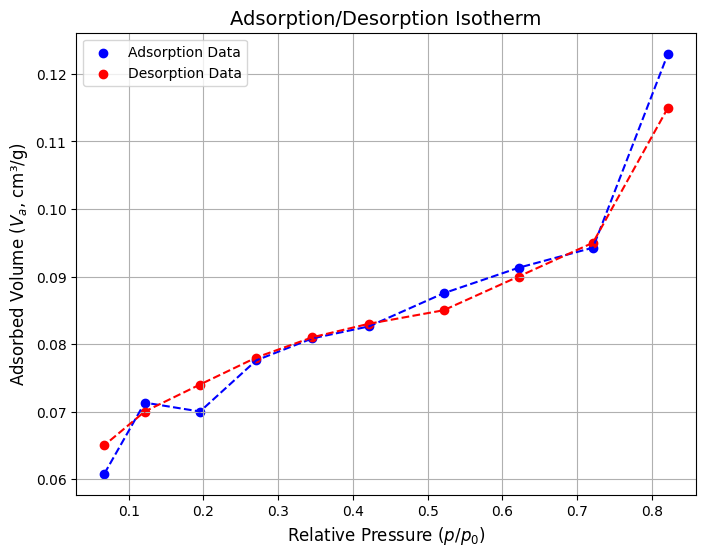

Supplement: Supplementary file 1 — Supplementary Material 1 [file 41598_2025_20677_MOESM1_ESM.docx]
